# The Relationship Between Entrepreneurial Intention and Action: The Effects of Fear of Failure and Role Model

**DOI:** 10.3389/fpsyg.2020.00229

**Published:** 2020-03-05

**Authors:** Fanzhu Kong, Lily Zhao, Cheng-Hung Tsai

**Affiliations:** ^1^Huaiyin Institute of Technology, Huai’an, China; ^2^Department of Business Administration, Cheng Shiu University, Kaohsiung City, Taiwan

**Keywords:** entrepreneurial intention, entrepreneurial behavior, business role model, fear of failure, China

## Abstract

The purpose of the study was to examine the moderating effect of business role model and fear of failure on the relationship between entrepreneurial intention and behavior. The participants were sampled from 1865 college students who graduated from universities in China from 2012 to 2018. The experimental instrument was comprised of four scales concerning entrepreneurial intention, entrepreneurial behavior, fear of failure, and business role model. The data were analyzed using hierarchical regression. The results showed that: (1) Entrepreneurial intention was positively influenced the entrepreneurial behavior; (2) Fear of failure weakened the relationship between entrepreneurial intention and action; (3) The moderating effect of business role model on entrepreneurial intention and behavior was confirmed. We concluded that entrepreneurial intention was positively related to the entrepreneurial behavior, fear of failure hindered college students from taking entrepreneurial behavior, and business role model will enhance their entrepreneurial intention. The paper suggest that some measures should be taken to overcome college students’ fear of failure, and to improve the education system of entrepreneurship in order to cultivate talents with creativity.

## Introduction

The process of entrepreneurship can be divided into two stages: the formation of entrepreneurship intention and the implementation of entrepreneurship behavior. Every entrepreneurship starts from the generation of entrepreneurship intention, which has a good predictive effect on entrepreneurship behavior. At present, many studies focus on entrepreneurship intention, they explored the factors affecting individual entrepreneurship intention from many aspects, such as personal characteristics, self-efficacy, risk perception, system design and so on. It is undeniable that the entrepreneurial intention is a necessary condition for the development of entrepreneurship. Relevant research results have a positive effect on our understanding of the entrepreneurship process. But can entrepreneurship behavior be guaranteed with entrepreneurship intention?

Liu et al. (2011) believed that people with entrepreneurial intentions may not really be able to start new businesses because of personal characteristics and the surrounding environment. That is to say, although entrepreneurship intention is a necessary condition for the occurrence of entrepreneurship, not all potential entrepreneurs can take action even if they want to start a business. Why do some people start businesses instead of others? What promotes or hinders the action of potential entrepreneurs? These are issues which deserve exploration and research. Relevant studies have shown that risk tendency, business role model, and entrepreneurial climate have a significant impact on individual entrepreneurship, but only focus on entrepreneurial intention, not extended to entrepreneurial practice. Therefore, fear of failure and business role model are chosen to explore the mechanism of individual entrepreneurial intention transforming into entrepreneurial behavior.

College students are an important part of mass entrepreneurship. Compared with other groups such as the new generation of migrant workers, college students have a more solid theoretical knowledge and advanced entrepreneurial concept, with active thinking and the spirit of adventure that they do not have, and with the gradual promotion of entrepreneurship education in schools, college students have a more comprehensive and profound understanding of entrepreneurship. As a result, College Students’ entrepreneurship has gradually broken through the scope of the original education, and has attracted more and more attention of scholars in other fields. They have studied it from the aspects of cultivation of entrepreneurship ability, construction of social environment, policy support, etc. However, they ignore the lack of social experience of college students and the fact that most Chinese students are less confident. Compared with other groups, it is more necessary to explore the role of positive psychology and role model in the process of college students’ entrepreneurship. The results of this study not only contribute to the entrepreneurship of college students in mainland China, but also provide an important reference for all Chinese groups in the world.

## Literature Review and Research Hypothesis

### Theoretical Foundation-Psychological Capital

Psychological capital first appeared in the economics. A person’s psychological capital is likely to govern their motivation and general attitude toward work. Goldsmith believes that Psychological capital is the integration of individual’s work, ethics, self-life belief, attitude and cognition, and some personality characteristics that can affect individual productivity (Goldsmith et al., 1997). Subsequently, psychological capital has attracted the attention of active psychologists and active organizational behaviorists. Seligman believes that the psychological factors that may lead to individual positive behavior should be included in the category of capital (Eligman and Csikszentmihalyi, 2000). Luthans and Youssef (2004) put forward the concept of psychological capital from the perspective of positive organizational behavior, and pointed out that psychological capital is a psychological state that can affect individual positive behavior. Psychological capital helps to regulate individual cognitive process, guide individual positive behavior process, and improve individual performance. Scholars selected job satisfaction, organizational commitment, coping style, interpersonal harmony, altruistic behavior, occupational well-being and mental health and other performance-related behavioral state variables to study the role and impact of psychological capital on these variables. The results showed that the effect of psychological capital on behavioral attitude was positive and significant, which can improve individual attitude and behavior by adjusting individual cognitive process, and ultimately affect individual performance (Du and Zhao, 2012).

Entrepreneurs with high psychological capital can balance the costs of failure better than those with low psychological capital, learn from failure and further manage and control enterprises effectively. According to the study, individuals with high psychological capital show a positive psychological state. When facing difficulties, they are more inclined to seek solutions to overcome problems. They can take failure as an excellent opportunity to learn, reflect on and summarize failure, and further improve to achieve their entrepreneurial goals (Zhuang, 2018). Therefore, psychological capital can correct entrepreneurs’ fear of failure. When encountering setbacks or hard work in the process of entrepreneurship, a successful entrepreneur can help them build a positive psychology of entrepreneurship, reduce the frustration of entrepreneurship, and then regain confidence (Chlosta et al., 2012; Tsai and Hsieh, 2012; Lafuente and Vaillant, 2013; Yang et al., 2013; Liu and Lin, 2015; Zapkau et al., 2015; Zampetakis et al., 2017; Bockorny and Youssef-Morgan, 2019; Wei et al., 2019).

### Entrepreneurial Intention and Entrepreneurial Behavior

DeNoble et al. (1999) holds that entrepreneurial intention is the entrepreneur’s intrinsic cognition, preference and behavioral tendency to create a new business. Krueger (2000) interpret entrepreneurial intention as a subjective attitude and expectation of potential entrepreneurs about whether they engage in entrepreneurship activities or not. Thompson (2009) defines entrepreneurial intention as the belief that entrepreneurs intend to start a business. In a word, entrepreneurial intention is a psychological state that guides our attention toward specific business goals in order to achieve entrepreneurial results. It is also a recognition that individuals take actions to develop new businesses or create new values in existing enterprises.

Entrepreneurial behavior is the process in which entrepreneurs put their entrepreneurial vision into practice. It is also the process in which entrepreneurs transform and create more wealth and value and achieve their entrepreneurial goals in a certain way by using the information, resources, opportunities or technologies they owned. Mcmullen and Shepherd (2006) argues that entrepreneurial behavior is an activity that entrepreneurs struggle to change and reach their goals by using their creativity and influence. Entrepreneurship is a combination of consciousness and planned behavior. It is a subjective state of entrepreneurs’ attention, energy and behavior oriented to a specific goal (Bird, 1988). The decision to build a new business is considered as a thoughtful thinking activity and planned behavior of entrepreneurs (Liu et al., 2011). Therefore, entrepreneurship ideas stimulated by inspiration must be reached through entrepreneurial intention. Entrepreneurial intention is the premise of entrepreneurial behavior. People with high entrepreneurial intention are more likely to start a new business than those with low. Based on this, this study proposes the following hypothesis:

Hypothesis H1: Individual entrepreneurial intention is positively correlated with Entrepreneurial behavior.

### Business Role Model and Entrepreneurial Behavior

A role model is a person who can influence others to some extent in social life. In social practice, individuals tend to look for similar examples. Individuals cannot participate in social activities in isolation, but act in groups with others. In this process, individual behavior decisions are often influenced by other people’s opinions and behaviors. Such “others” are often referred to as “role models” (Gibson and Barron, 2003). Whether “others” can become “role models” is related to your perception. When you think that you are somewhat similar to “others” and therefore tend to imitate or deliberately avoid certain attributes or behaviors of “others,” “others” become your “role models” (Bosma et al., 2012).

As the carrier of social learning, role model provides individuals with a reference to learn experience. Through observational learning of role models, individuals can acquire successful or unsuccessful experience of role models, and form clear self-judgments about certain attributes or behaviors of successful imitation or avoidance of role models in similar situations. In addition, the role models can directly affect individuals by involving them in learning activities.

For entrepreneurship, the role of business models is to provide individual spiritual incentives and behavioral guidance, which has an important impact on individual entrepreneurship activities (Bosma et al., 2012). Krueger’s research shows that business role models play an important role in the career decision-making process in which individuals choose to become entrepreneurs. As far as potential entrepreneurs are concerned, business role models can provide indirect experience such as business information, improve the probability of discovering and utilizing business opportunities, and then enhance the possibility of triggering entrepreneurship events. That is to say, if your relatives or friends are entrepreneur, you may also start a business. Li et al. (2017) takes farmers as the research objects, empirically studied the relationship between business role model and entrepreneurial behavior. The results showed that the farmers with business role model are more likely to trigger entrepreneurship events than those others. Based on the above discussion, this paper puts forward the following hypotheses:

Hypothesis H2: business role model is positively correlated with entrepreneurial behavior.

### Fear of Failure and Entrepreneurial Behavior

Fear of failure develops gradually from the study of achievement motivation. Avoiding failure is an instinctive behavior of people. Birney et al. (1969) believes that fear of failure refers to the inner fear of an individual when he or she perceives that he or she may not achieve certain goal. The fear of failure is the opposite of the desire to succeed and the motivation to avoid punishment for failure. For most people, fear of failure is uncontrollable. Individuals are often affected by fear of failure and adopt the behavior of avoiding achieving goals, if he can’t be guaranteed success (Ajzen, 1991).

Fear of failure is closely related to uncertainty and risk aversion. Lipshitz and Strauss (1997) argues that uncertainty is a kind of sensory doubt that can hinder or delay the occurrence of behavior. Therefore, uncertainties in the process of entrepreneurship can lead to hesitation and procrastination, which is so damaging to entrepreneurial behavior. In addition, risk aversion may also be a manifestation of fear of failure. It is believed that the more risk-averse a person is, the more afraid he is of failure. In the process of entrepreneurship, entrepreneurs are more inclined to choose entrepreneurial behavior with moderate risk or even zero risk (Ekore and Okekeocha, 2012).

Both professionals and college students, the fear of failure may become an obstacle to their entrepreneurship. In general, employees have formed a relatively stable living condition. For them, entrepreneurship means changing the original rhythm of life. Once failed, it will not only be unable to maintain the original living standard, but may also be in a dilemma. This will invisibly delay or put aside the entrepreneurship ideas of the incumbents. The results showed that fear of failure had a negative impact on female entrepreneurs. Liu et al. (2011) argued that some potential entrepreneurs would not start a business because they were afraid of various risks brought about by business failure. Entrepreneurship not only needs to raise funds, human resource and equipment during the preparatory period, but also faces many challenges and crises at any time. Considering their abilities and possible risk of failure, the number of people who really start a business may be greatly reduced. Accordingly, this study proposes the following hypotheses:

Hypotheses H3: Fear of failure is negatively correlated with Entrepreneurship behavior, and negatively regulates the relationship between individual entrepreneurial intention and entrepreneurial behavior.

### Business Role Model and Fear of Failure

The role of the role models has been extensively studied in different fields such as sociology and psychology. Child development psychology has proved that role models can help children overcome fear; even adults, positive role models can alleviate their social fears, which results from imitative learning of role models. As far as entrepreneurship is concerned, business role models set a benchmark for individuals. Through social comparison, observation Learning and imitation learning, potential entrepreneurs deepen their understanding of entrepreneurship projects similar to those experienced by business role models. They have a more intuitive and accurate understanding of the resources needed for entrepreneurship and the problems that may arise in the process of entrepreneurship, which can eliminate the anxiety and fear of entrepreneurship.

As for the relationship between role models and overcoming fear, it seems that we can also draw inspiration from the children’s story of “Pony across the river.” After receiving his mother’s instructions, the pony happily went to the village with his hunched grain. On the way to the river, he dared not cross the river because he did not know the depth of the river. Even if he had acquired the external experience knowledge such as cow and squirrel, he could not rule out the fear of crossing the river. So, he went home to ask for his “role model” mother’s advice. With his mother’s encouragement, the pony fight down the fear and successfully crossed the river. In this story, the pony gradually overcomes the fear of the river and finally reaches the other side of the river based on his trust in the “role model.” Relevant studies in the field of entrepreneurship also believe that business role models can not only provide entrepreneurship information, but also provide spiritual and material support. These supports reduce many “worries” for potential entrepreneurs, strengthens their confidence in overcoming difficulties in the process of entrepreneurship, and improves the positive evaluation of the possibility of success of entrepreneurship. Although the above views have not been proved by empirically studied, but at least to show that there is a link between business role models and fear of failure. Based on the above discussion, this study put forward the following hypotheses.

Hypotheses H4: Business role models are negatively correlated with fear of failure. Business role models could help potential entrepreneurs overcome fear of failure.

**FIGURE 1:**
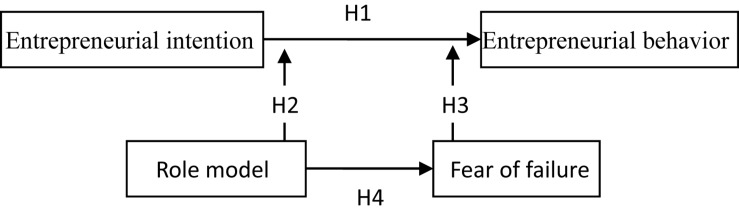
Research framework.

These four hypotheses constitute the framework of this study, as shown in Figure 1.

## Research Methods and Data Collection

### Operational Definition of Variables

Therefore, in this study, the “yes” and “no” of each variable are assigned to “1” and “0,” respectively, for correlation analysis and regression analysis.

#### Entrepreneurial Intention

This paper drew lessons from this item, and changed the topic for the respondents. The revised topic is: Do you expect to start a business one day when you graduate from university? Answer “yes” on behalf of their entrepreneurial intention, on the contrary, they do not have entrepreneurial intention, and “yes” to “1,” “no” to “0.”

#### Entrepreneurial Behavior

The measurement of entrepreneurial behavior has always been a difficult problem in the academic community, which is very challenging, but also an important issue to be solved in the process of entrepreneurial research. The initial entrepreneur plays a key role in the process of enterprise creation is. Although there are few definitions, the initial entrepreneur is always regarded as a person if he starts a series of behaviors at first, and if the intention is to create the enterprise at last, that is the entrepreneurial behavior (Aldrich and Martinez, 2001). Four related items were designed. Each item was answered in the form of “yes or no” (yes was given a value of “1” and “no” was given a value of “0”). After that, the selection scores of the four items were added up, with the highest score of 4 and the lowest score of 0. The entrepreneurial behavior was measured by this score. The higher the score, the higher entrepreneurial behavior. This study also uses this way to measure entrepreneurial behavior, just the words of the title have been revised, so as to make it more in line with Chinese vocabulary habits. The revised topics include “Are you currently trying to start a business,” “From graduation to now, have you participated in or invested in other people’s business” and so on.

#### Fear of Failure

Fear of failure is used to measure whether an individual will give up the idea of starting a business because he was afraid to fail. The answer “No” means that they will not be held back by fear of failure, and the “yes” to the value of 1, “no” to the value of 0.

#### Business Role Models

Business role models are used to measure whether individual entrepreneurship decisions are influenced by role models. This paper also uses this form to set up “Are there successful entrepreneurs among your family, teachers, relatives and friends?” The answer “Yes” means that they are influenced by business role models, “No” means that they are not affected by role models, and the “yes” value is given to 1, and the “no” value is given to 0.

#### Control Variables

Previous studies have shown that the entrepreneurial intention of males is significantly higher than that of females, and individuals with different professional backgrounds and family economic conditions have different entrepreneurial intention. Therefore, in order to avoid these factors affecting the role model effect of this study, gender, major and household income were taken as control variables.

### Data Collection

The main purpose of this paper is to explore whether business role models and fear of failure have an impact on individual entrepreneurial behavior. According to two-stage model of entrepreneurship (Mcmullen and Shepherd, 2006), the problem studied in this paper belongs to the transition stage of entrepreneurship, so we choose graduates as respondents, which can not only avoid the errors caused by the retrospective approach, but also meet the needs of research purposes. We selected more than 1800 students who graduated from 35 universities in China from 2012 to 2018 and sent questionnaires by e-mail and QQ group. A total of 1865 questionnaires were sent out, 769 were actually recovered. After eliminating the invalid questionnaires, 698 valid questionnaires were finally obtained. The descriptive statistical results of the samples are shown in Table 1. According to Standards for Classification of Family Income in China in 2015, the paper divided household income into six categories.

**TABLE 1:** Variable description.

**Variable**	**Sample number**	**Proportion (%)**
**Sex**		
male	426	61.03
female	272	38.97
**Specialty**		
Economic management	201	28.80
Humanities and social sciences	146	20.92
Science and engineering	312	44.70
Agriculture cure	39	5.58
**Household income (CNY/Year)**		
<50 thousand	12	1.73
50–100 (50 ≤ , <100, the same below) thousand	74	10.60
100–200 thousand	221	31.66
200–300 thousand	212	30.37
300–1000 thousand	145	20.77
≥1000 thousand	34	4.87

Table 1 shows that 61.03% of the students are male, 44.70% in science and engineering, 28.80% in economy and management, and only 5.58% in agriculture and cure. The distribution of household income is almost in line with China’s household income statistics, with 82.9 percent of the total number of households in the well-off and middle-income categories.

## Analysis of Research Results

### Reliability and Validity Test

Reliability analysis, also known as reliability testing, is used to test the reliability of collected data. At present, most scholars use Cronbach’s α as an indicator to test the reliability, and its value is greater than 0.7 to be considered as having a good reliability. This paper also uses this method to analyze the reliability of entrepreneurial behavior. The results show that the Cronbach’s α of the variable is 0.872, and the factor load of each item is above 0.6, which indicates that the scale of entrepreneurial behavior has good reliability.

Validity usually includes content validity and structure validity. Because the measurement of variables in this paper is based on more mature scales used in European and American countries, it can be considered that it has a good content validity. The detection of structural validity is usually based on factor analysis. Since most of the variables involved in this paper are single-dimensional variables, factor extraction is not necessary. Therefore, this paper only analyzes the validity of the variable of entrepreneurial behavior. Before factor analysis, the KMO value and Bartlett spherical test were used to test whether the samples were suitable for factor analysis. The results showed that the KMO value was 0.796, the chi-square value was 892.327, and the significance probability was significant at the level of 0.05, which indicated that the data were correlative and suitable for factor analysis. The test results showed that a common factor is extracted, and the cumulative variance of the factor reaches more than 60%, which is more than 50% of the basic standard. It can be considered that the scale of entrepreneurial behavior has good structural validity.

### Relevance Analysis

Using SPSS20.0, Pearson correlation coefficient was used to analyze the correlation between various variables. The results are shown in Table 2. There was a significant positive correlation between entrepreneurial intention and entrepreneurial behavior, and there was no significant correlation between fear of failure and entrepreneurial intention, but there was a significant negative correlation between fear of failure and entrepreneurial behavior and business role model. There was a significant positive correlation between business role model and entrepreneurial intention and entrepreneurial behavior, but the value of correlation between business role model and entrepreneurial intention was significantly greater than that between business role model and entrepreneurial behavior. After analyzing the correlation between fear of failure and other variables, we analyzed the correlation between fear of material loss, fear of mental loss and entrepreneurial intention, entrepreneurial behavior, and business role model. The results showed that fear of material loss has a significant negative correlation with entrepreneurial intention, entrepreneurial behavior and business role model. The relationship between the fear of mental loss and entrepreneurial intention is not significant, but it is significantly negatively related to entrepreneurial behavior and business role model. In the correlation statistics, men we give the value 1, women give the value 0, and household income is given the value 1–6 in a low to high order.

**TABLE 2:** Sample correlation.

**Variable**	**1**	**2**	**3**	**4**	**5**	**6**	**7**
1 Sex	1						
2 Specialty	0.03^∗^	1	1	1			
3 Income	0.01	0.01	0.03				
4 Intention	0.042	0.10^∗^					
5 Behavior	0.04^∗^	0.09^∗^	0.10^∗∗^	0.33^∗∗∗^	1		
6 Fear	0.02	0.04	–0.12^∗∗^	–0.03	–0.26^∗∗∗^	1	
7 Role model	0.01	0.02	0.006	0.46^∗∗∗^	0.13^∗^	–0.15^∗∗^	1

*The diagonal number is the square root of average variation extraction, ^∗∗∗^*p* < 0.001, ^∗∗^*p* < 0.01, ^∗^*p* < 0.05.*

### Hypothesis Test

#### Relationship Between Entrepreneurial Intention and Entrepreneurial Behavior

The correlation of Table 2 shows that the correlation coefficient between entrepreneurial intention and entrepreneurial behavior is 0.326, reaching a significant level of 0.001, indicating that entrepreneurial intention and entrepreneurial behavior are significantly positively correlated. Relevance only indicates that there is a certain correlation between them, but it cannot explain the action. In order to study whether entrepreneurial intention can predict entrepreneurial behavior, this study uses entrepreneurial behavior as dependent variable and entrepreneurial intention as independent variable to make regression analysis. The results are shown in Table 3, model 2. The study found that the relationship between entrepreneurial intention and entrepreneurial behavior is very significant, with a standardized coefficient of 0.321, indicating that entrepreneurial intention has a significant positive relationship with entrepreneurial behavior, Hypotheses H1 has been verified.

**TABLE 3:** Regression role model.

	Entrepreneurial behavior			
Variable	Role model 1	Role model 2	Role model 3	Role model 4
**Control variable**				
Sex	0.01	0.01	0.02	0.02
Specialty	0.08	0.08	0.07	0.07
Household income	0.15^∗^	0.12^∗^	0.10^∗^	0.07
**Independent variable**				
entrepreneurial intention		0.31^∗∗∗^	0.24^∗∗^	0.12^∗^
**Moderating variable**				
Fear of failure			–0.29^∗∗^	−0.17^∗^
Business role model			0.01	0.02^∗^
**Product-item**				
entrepreneurial intention and fear of failure				–0.35^∗∗∗^
entrepreneurial intention and business role model				0.18^∗∗^
*R*^2^	0.03	0.09	0.11	0.10
*Adj R^2^*	0.02	0.06	0.12	0.13
*ÄR^2^*	0.02	0.08	0.17	0.13
*F-Value*	22.18^∗∗∗^	41.52^∗∗∗^	66.67^∗∗∗^	58.26^∗∗∗^

*^∗^*p* < 0.05; ^∗∗^*p* < 0.01; ^∗∗∗^*p* < 0.001.*

#### Moderating Effect of Business Role Model and Fear of Failure

This study used hierarchical regression analysis to test the moderating effect of business role model and fear of failure, and regarded gender, specialty and household income as the control variables, entrepreneurial intention as the independent variable, entrepreneurial behavior as the dependent variable, business role model and fear of failure as the moderating variables. Considering the effects of control variables, the hierarchical regression analysis to test the moderating effect generally consists of four steps: The first step is the control variable model, the second step is the model with the independent variable, the third step is the model with the moderating variable, and the fourth step is the model with the interaction term of the independent variable and the moderating variable.

Firstly, the paper tested the control variables model (Table 3, Role model 1). The results showed that the model fits well, and the regression weights of gender and specialty are not significant. However, household income has a significant effect on entrepreneurial behavior, indicating that the level of family income may affect whether college students with entrepreneurial intention engage in entrepreneurship action. Secondly, the paper tested the moderating variable model (Table 3, Role model 3). The results showed that the model fits well and that fear of failure has a significant effect on entrepreneurial behavior, but the effect of business role model is not significant, indicating that business role model may not have a direct impact on entrepreneurial behavior. Thirdly, we put the product-item of independent variables and moderating variables into the model (Table 3, model 4). In order to avoid co-linear between independent variables and interactive terms, the product terms of independent variables and moderating variables are averaged. From Table 3, role model 4, we can see that the product-item of entrepreneurial intention and business role model has significant positive effect on entrepreneurial behavior. According to the above, Hypotheses H2 is supported. At the same time, it could be concluded that product-item of entrepreneurial intention and fear of failure has significant negative effect on entrepreneurial behavior from Role model 4, Hypotheses H3 was supported.

#### The Relationship Between Business Role Model and Fear of Failure

According to the sample correlation in Table 2, the correlation coefficient between business role model and fear of failure is −0.15, which reaches a significant level of 0.01, indicating that business role model is negatively correlated with fear of failure. Then, this study regarded business role models as independent variables and fear of failure as dependent variables for regression analysis. The results showed that the relationship between business role models and fear of failure is very significant, and the standardization regression weight is −0.25. It shows that business role models have a significant negative effect on fear of failure. That is to say, the business role models would help potential entrepreneurs overcome fear of failure. Hypotheses H4 has been verified.

## Conclusion and Discussion

### Theoretical Contribution

The purpose of this study is to explore the transformation mechanism of individual entrepreneurial intention to entrepreneurial behavior, and explain the role of business Role model and fear of failure in the process. Most studies have supported the view that entrepreneurial intention is the best predictor of entrepreneurial behavior. However, they almost stay at the stage of theoretical discussion, or directly regard this view as the presupposition basis of research, while there are few empirical studies. This study takes Chinese university graduates as a special group to study the relationship between entrepreneurial intention and entrepreneurial behavior. In order to ensure the reliability of the survey results, the questionnaires were sent and taken back by the teacher in charge of the class through e-mail, class group, etc. Questionnaires are distributed to many universities in different regions, which ensures the diversity of sample sources. The results showed that individual entrepreneurial intention has a positive impact on their entrepreneurial behavior. That is to say, when individuals have a higher entrepreneurial intention, the more likely they are to carry out entrepreneurial behavior. This empirical research conclusion not only supports the existing theoretical presupposition, but also indirectly shows that although culture influences individual behavior, the phenomenon that intention is the premise of individual behavior is common to different cultures (even if it cannot exclude the occasional behavior).

The business role model positively regulates the relationship between entrepreneurial intention and entrepreneurial behavior. Most of the existing studies focus on the impact of entrepreneurial role models on entrepreneurial intention, less on entrepreneurial behavior, while this study tried to find out the relationship between business role model and entrepreneurial behavior. From the results of sample correlation, business role models are significantly positively correlated with entrepreneurial intention and entrepreneurial behavior, but the correlation coefficient with entrepreneurial intention is significantly greater than that with entrepreneurial behavior, which may indirectly indicate that the impact of business role models on individual entrepreneurship is mainly focused on willingness motivation. Regression analysis also showed that the regression weight of business role model on individual entrepreneurial behavior is not significant. Based only on the result, business role model may not have a direct effect on entrepreneurial behavior. From the interaction effect between business role model and entrepreneurial intention, the regression weight of entrepreneurial intention on entrepreneurial behavior decreases after adding the moderating variable of business role model, while the regression weight of business role model on entrepreneurial behavior changes from insignificant to significant, and the interaction effect is significant, which showed that business role model plays a positive moderating role between entrepreneurial intention and entrepreneurial behavior. According to the research on the relationship between business role models and entrepreneurial intention, it seems that entrepreneurial intention plays a part of intermediary role between business role models and entrepreneurial behavior. That is to say, business role models not only positively affect entrepreneurial intention, but also indirectly affect entrepreneurial behavior through the intermediary role of entrepreneurial intention, which needs to be explored in future research.

Fear of failure negatively regulates the relationship between entrepreneurial intention and entrepreneurial behavior. To answer the question, “Why do some people, not others, take entrepreneurial actions?” This study also introduced the fear of failure as a variable. The sample correlation showed that the correlation between fear of failure and entrepreneurial intention is not significant, but it is significant with entrepreneurial behavior, and the regression weight also reaches a significant level. This indicated that fear of failure has a negative effect on entrepreneurial behavior. That is to say, the more you worry about failure, the less you dare to start a business. Therefore, helping potential entrepreneurs overcome the fear of failure can increase the probability of their entrepreneurial behavior. In terms of the dimension of fear of failure, the fear of material loss is stronger than the fear of mental loss. This may indicate that the would-be entrepreneurs think first of all about making money, then reputation and self-realization.

Child development psychology has proved that role models can effectively overcome children’s fear. Can this research result extend to the field of adult entrepreneurship? In order to answer this question, this study proposed and tested the hypothesis that business role models are negatively correlated with fear of failure. From the results, there is a significant negative correlation between business role models and fear of failure, and the regression weight has reached a significant level, indicating that successful business role models could help individuals overcome fear of failure, and then stimulate their entrepreneurial behavior. Positive psychology advocates that human beings should face many psychological phenomena with a positive mentality, and use it to stimulate some actual or potential positive qualities and positive forces inherent in each person, so that everyone can move toward their own happiness. Business role models may help stimulate the positive forces of individuals, thereby eliminate negative perceptions such as fear, and ultimately adopt entrepreneurial behavior.

According to the theory of social action, structure determines action, and the elements of structure are various. Different structural elements have different effects, and there may be cross-interaction among them. As far as entrepreneurial behavior is concerned, its structural factors are far beyond the business role model and fear of failure, at least including the identification of business opportunities, access to entrepreneurial resources and other factors. This paper only examines the impact of business role models and fear of failure on entrepreneurial behavior. It didn’t consider the role of other factors, nor consider the possible cross-interaction between business role models and fear of failure and other factors. This range of issues need to be studied in the future.

### Practical Enlightenment

Set up role model and stimulate the entrepreneurial willingness of College Students. The role model has a subtle influence on the entrepreneurial willingness of college students, so colleges and universities and the government should pay attention to the role of entrepreneurial models. Role models have a very good incentive effect. When college students see that someone around them is successful in starting a business, their self-confidence will be significantly enhanced, and their passion for starting a business will be significantly improved. Therefore, colleges and universities should strengthen the publicity of entrepreneurial models, invite successful entrepreneurs to the school to share their experience in starting a business, and let students get a chance through observing, learning and imitating the entrepreneurial process of role models Connect with entrepreneurial experience, and then enhance entrepreneurial self-efficacy and entrepreneurial willingness. Secondly, the government should make full use of newspapers, TV, Internet and other ways to publicize the entrepreneurial model, improve the public’s recognition of entrepreneurship, create a strong entrepreneurial atmosphere that encourages entrepreneurship, supports entrepreneurship and tolerates failure, and stimulate the entrepreneurial willingness of college students.

Create a good entrepreneurial environment, reduce the fear of failure of college students’ entrepreneurs. Some college students who are willing to start their own business, but because of lack of entrepreneurial experience and resources, they are afraid of failure and delay or avoid taking entrepreneurial behavior. Therefore, the government and colleges and universities should strive to create a good entrepreneurial environment, provide more preferential entrepreneurial policies for college students, give entrepreneurial fund support, establish a complete entrepreneurial insurance system, and other policies to eliminate college students’ worries about entrepreneurship as much as possible. So as to reduce the entrepreneurial failure of college students. They can objectively understand entrepreneurial activities, accurately identify and grasp entrepreneurial opportunities, and then improve the proportion of entrepreneurship.

Strengthen the psychological quality education of college students. The process of entrepreneurship is long, complex and hard. In the process of entrepreneurship, there will be many unexpected difficulties. This requires that potential college students’ entrepreneurs not only have certain entrepreneurial knowledge and skills, but also have good entrepreneurial psychological quality, so as to cope with the complex and changeable situation in the process of entrepreneurship. Therefore, colleges and universities should strengthen the psychological quality education of students, through the entrepreneurial psychological workshop, group entrepreneurial psychological guidance, personalized psychological guidance, psychological mutual assistance of friends and elders, willpower training, environmental tolerance and psychological adaptability guidance, so that college students will be more rational and confident on the road of entrepreneurship in the future, and less detours.

## Data Availability Statement

The datasets generated for this study are available on request to the corresponding author.

## Ethics Statement

Ethical review and approval was not required for the study on human participants in accordance with the local legislation and institutional requirements. Written informed consent for participation was not required for this study in accordance with the national legislation and the institutional requirements.

## Author Contributions

FK wrote the original manuscript. LZ collected and analyzed the data. C-HT reviewed the manuscript and validated the all research procedures.

## Conflict of Interest

The authors declare that the research was conducted in the absence of any commercial or financial relationships that could be construed as a potential conflict of interest.
